# Costing of diabetes mellitus type II in Cambodia

**DOI:** 10.1186/s13561-014-0024-4

**Published:** 2014-11-01

**Authors:** Steffen Flessa, Anika Zembok

**Affiliations:** Department of Health Care Management, University of Greifswald, Friedrich-Loeffler-Straße 70, Greifswald, 17487 Germany

**Keywords:** Cambodia, Costing, Diabetes, Markov-Model, Prediction, South-East Asia, T2DM

## Abstract

**Background:**

Diabetes Mellitus Type II (T2DM) is a major and growing medical, social and economic burden in the East-Asian country of Cambodia. However, no economic modelling has been done to predict the number of cases and the budget impact.

**Objective:**

This paper forecasts the epidemiological and economic consequences of T2DM in Cambodia. The Ministry of Health and related donor agencies are supported to select the most cost-effective interventions against the disease. At the same time this paper demonstrates the relevance and potential of health economic modelling for least developed countries.

**Methods:**

We developed a Markov-Model for the specific situation of Cambodia. Data was taken from the scientific literature, grey literature in Cambodia and key-informant interviews.

**Results:**

The number of people living with T2DM is steadily increasing from 145,000 in the year 2008 to 264,000 in the year 2028 (+82 %). In the year 2008 the diagnosed T2DM patients would incur costs of some 2 million US$ to cover all of diabetes treatment. 57 % of this amount would have to be spent for OAD-therapy, the rest for insulin therapy. In the year 2028 this amount will have grown to some 4 million US$. If all patients (incl. non-diagnosed) had to be paid-for the respective figure would be 5.5 million and 11 million US$.

Screening for T2DM is only cost-effective if the sensitivity of the test is high while the unit price is low. The results of this simulation call for targeting the high-risk groups. However, an increased availability of Oral Anti-Diabetic and Insulin Therapy is highly cost-effective.

**Discussion:**

Type 2 Diabetes Mellitus is a major public health challenge in Cambodia. The simulations clearly indicate that prevention and treatment of this disease is highly cost-effective. However, not everything that is cost-effective might be affordable in Cambodia. This country will require external support to ease the growing burden of T2DM.

## Background

Diabetes Mellitus Type II (T2DM) is a non-communicable disease (NCD) with a high prevalence and an increasing incidence worldwide. The International Diabetes Federation (IDF) estimates that about 350 million people are affected worldwide and some 90% of diabetes patients live in low- or middle-income countries [1]. IDF also expects a strong increase of diabetes cases which will result in tremendous human suffering, death cases and enormous costs of treatment [2]. Thus, T2DM is a disease of high public health importance and a good representative to analyse the economic impact of NCDs on the health care system of Cambodia and other low-income countries.

In the year 2011 diabetes was top-8 cause of death worldwide with 1,392,000 death cases (2.6% of all death cases worldwide) and a rate of 20 per 100,000 inhabitants. For South-East Asia (and the Western Pacific) WHO Region the respective figures are rank 8 (8), 388,000 (284,000) death cases, 2.8% (2.4%) of all death cases) and a rate of 21 (16) per 100,000. In the year 2000 diabetes had been only on rank 10 worldwide and in the two target regions of this paper [3]. Thus, diabetes is of high public health relevance. It is one of the most prominent NCDs and a major cause of morbidity and mortality worldwide as well as in South-East Asia and the Western Pacific Region.

The South-East Asian country of Cambodia has a specific historical pathway that led to a late start of the epidemiological transition and a specific age structure. The population pyramid of 1990 shows the dramatic impact of the Khmer Rouge period (1975–79). However, the population of Cambodia is rapidly aging. As chronic-degenerative diseases are mainly age-determined [4], it is extremely likely that the absolute and relative importance of NCDs such as T2DM is going to increase in Cambodia.

Currently the population is still rather young so that the share of morbidity and mortality caused by NCDs such as diabetes is slightly lower than in other countries of the region. The STEPS-Survey 2010 [5] records a prevalence rate of T2DM of the adult Cambodians (25–64 yrs.) of 2.9% (170,000 people), but there are strong diversions between age groups, regions and gender. Younger adults (23–34 yrs.) have a lower prevalence rate (1.1%); older groups (55–64 yrs.) a higher rate (6%). The prevalence rate of the urban population (5.6%) is much higher than of the rural population (2.3%). At the same time, women (3.3%) are more affected than men (2.5%). Other surveys [6] come to slightly different figures but confirm the general finding that T2DM is a major public health problem in Cambodia.

According to WHO, Diabetes causes 3,122 death cases (2008) and a loss of 39,000 disability adjusted life years (DALY) in Cambodia per year (2004, latest up-date of WHO burden of disease database) [7]. IDF records 5,540 death cases in 2013 [8]. However, to our knowledge no estimate of the economic cost of T2DM from a society point of view was attempted. However, this disease is the fourth important NCD cause of death and the sixth importance NCD cause of loss of quality of life in this country.

Prevention and treatment of T2DM in Cambodia is limited as the public health services do hardly provide any diabetes care, T2DM is not included in the “basic package of health care” and oral anti-diabetic drugs (OAD) and insulin are not available in the whole country. The Royal Government of Cambodia wants to address this rising disease and take care of its population, but for this purpose it urgently needs a prediction of the expected number of T2DM cases in Cambodia, determine the most cost-effective intervention strategies, and estimate the budget impact of prevention and treatment of this disease.

Unfortunately, no economic modelling of T2DM was ever done in Cambodia. There are some cost estimates from other South-East Asian countries, such as Indonesia [9-11], Malaysia [12,13], Philippines [14-16], Thailand [17] and Vietnam [14] but these cannot easily be transferred to Cambodia with its unique age structure, economic situation and age structure. A very rudimentary costing comes from the World Diabetes Foundation [18] which run a project of diabetes care which cost 15.70 US$ per patient annually or 6.00 US$ per consultation with all treatment measures. Raguenaud et al. [19] estimated 48 US$ per year (p. a.) for a glibenclamide mono-therapy and 192 US$ p. a. of bi-therapy with glibenclamide and metformin. However, these figures are based on a pilot project of limited scope and geographical extent. They cannot be taken as a standard for a public health intervention in the entire country under the wings of the Ministry of Health.

Thus, our knowledge of the economic burden of T2DM in Cambodia is as limited as our insights into the cost-effectiveness of certain interventions in this country. There is a great need to base decisions of the Ministry of Health of the Royal Government of Cambodia as well as of the international health partners on evidence. Consequently, we have to make a micro-costing, a budget-impact and a cost-effectiveness analysis of interventions against T2DM. For this purpose we developed a Markov model of T2DM in Cambodia which will be presented in the next section. The results (to follow in section three) build the foundation of the Annual Operational Plan of the Ministry of Health in Phnom Penh. Consequently, our analysis takes the perspective of the Ministry of Health of Cambodia, but the insights are of relevance to other least developed countries in particular in the South-East Asian region.

## Methods

### Context

Cambodia is a least developed country with a gross domestic product of about 1,000 US$ (gross domestic product in current US$ per capita in 2013) [20]. Although the situation has improved within the last few years, still some 20% of the population is still below the official poverty level permitting hardly to survive daily life.

The Ministry of Health finances first-line (health centres) and second-line (hospitals) health facilities. However, patients have to pay modest fees. The services of these facilities are defined by the Ministry of Health as a so-called “basic package of health care services” [21]. Currently, this package includes only prevention and treatment of communicable diseases as well as diagnosis related to maternity. Due to financial constraints, NCDs are not included in this package although it is generally accepted that they have a high priority [22]. Cambodia spends about 50 US$ per annum per capita [20]which is not enough to include chronic-degenerative diseases in the basic package.

The population under the poverty-line is exempted from paying the fees by a system called “Health Equity Fund” (HEF). The health care provider receives the equivalent amount from the HEF which is partly financed by the international donor community and the Royal Government of Cambodia. However, if a patient suffers from a disease which is not covered by the basic package (such as diabetes) he has to seek services from private providers who usually charge fees unaffordable for the majority of the population. Thus, very limited treatment is available for the majority of diabetic patients.

Most diabetic patients are undiagnosed until they have severe complications as no blood-sugar testing is usually performed in health centres. As anti-diabetic drugs are frequently only available in private pharmacies for high prices, even those who are diagnosed will receive proper treatment only if they can afford it. The same is true for insulin. Consequently, self-help groups and “peer-educator” groups [23] have been started. MoPoTsyo (Patient Information Centre), for instance, supports diabetes patients with drugs and insulin. At the same time it teaches them to become peer educators for their villages. This includes the search for potential diabetic cases. Peer Educator Networks have been accepted by the Government as an important contribution to the fight against NCDs [22], but until now no funds are allocated by the Ministry so that most Cambodians remain without any support if they develop diabetes.

### Model

The analysis follows the standards of health economic evaluation stipulated by Schulenburg et al. [24] and takes the perspective of the Ministry of Health of Cambodia. The calculation and prognosis of the costs of different interventions against T2DM is of high importance for this Ministry. In addition, the Ministry of Health is obliged to include only new interventions into the basic package of services if they are cost-effective in comparison to other possible interventions. Thus, we also calculate the incremental cost effectiveness ratio (ICER) as this is a possible statistic that allows comparing the cost-effectiveness of possible candidates to be included into the basic package.

As diabetes is an NCD the likelihoods of developing diabetes or transitioning to another stage of the disease are constant. Consequently, the epidemiology of T2DM is frequently simulated with Markov Models [25]. However, only few models were designed for resource-poor countries [26]. None represents the specific structure and parameters of the Cambodian demography and the health care system. Thus, it was necessary to design a specific model and search for the relevant parameters of Cambodia.

**Figure 1:**
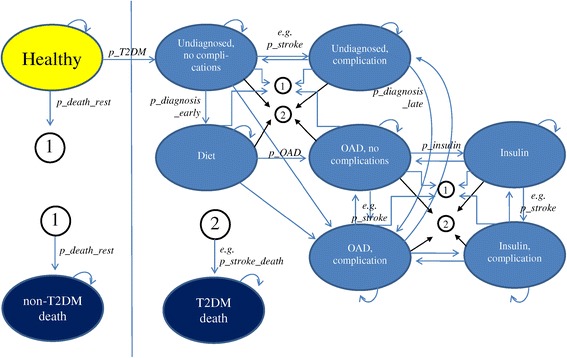
**Markov Graph (variables refer to (Table** 1 **)).**

The model simulates age-sets of 5 years for adults (>15 yrs.). The simulation runs from 2008 to 2028. As the prevalence of T2DM in the age set < 35 is negligible, we concentrate on the cohort ≥ 15 years in the year 2008. Figure 1 shows the principle structure of the Markov model recalling the biological life of a T2DM patient. Every year 1/5^th^ of the population is transferred to the higher age-set. At the same time, a certain part of the healthy population will die. The parameter p_death_rest is the non-T2DM related rest mortality for a certain age set. Another part of the healthy population will develop uncomplicated (and undiagnosed) diabetes. The respective share is denoted as p_T2DM and is age-specific for each age-set.

An undiagnosed diabetic case without complications can either stay in this condition or develop complications. For each complication (diabetic nephropathy, retinopathy, neuropathy, angina pectoris, peripheral vascular disease (PVD), myocardial infarction, stroke, heart failure) the respective transition probabilities (e.g. p_retino; p_neuro; p_stroke…) are applied. The patient can also die from diabetes (e.g. p_stroke_death) or non-diabetes (p_death_rest).

If the patient is diagnosed (with a likelihood of p_diagnose_early) he will be under diet. Again, the patient can stay in this compartment, develop complications, receive OAD or die from diabetes or any other cause. The respective transition probabilities are given in the (Table 1). Any patient under anti-diabetic drugs can stay in this compartment, develop a need for insulin injections, develop complications or die with certain likelihoods.

**Table 1:** **Basic parameters**

**Parameter**	**Explanation**	**Value**	**Source**
p_death_rest	non-T2DM mortality	age-specific	[27]
p_T2DM	age-specific incidence of T2DM	age-specific	[28]
p_Diagnosis_late	probability of clinical diagnosis with persisting complications	0.2	Assumption
p_Diagnose_early	probability of clinical diagnosis without complications or in the first year after development of complications	0.08	Assumption
p_OAD	probability of change of therapy to OAD-therapy	0.98	Assumption
p_Insulin	probability of change of therapy to OAD/insulin or mono-insulin	0.04	[29]
p_Insulin_Adherence	share of patients taking insulin in those requiring it	0.125	[30]
p_OAD_Adherence	share of patients taking OAD medication in those requiring it	0.125	[30]
p_stroke_death	stroke-related mortality	0.38136	[31]
p_MI_death	myocardial infarction-related mortality	0.7068	[31]
p_nephro	incidence diabetic nephropathy	0.01479	[32]
p_retino	incidence diabetic retinopathy	0.02124	[33]
p_neuro	incidence distal symmetric neuropathy	0.04658	[34]
p_Angina	incidence angina pectoris	0.00668	[31]
p_PVD	incidence PVD	0.00847	[35]
p_MI	incidence myocardial infarction	0.01735	[31]
p_stroke	incidence stroke	0.00529	[31]
p_heartfailure	incidence heart failure	0.00329	[31]
c_Diagnosis	unit cost FPG-test	1.00 USD	[36]
c_ambulatory	Annual cost outpatient treatment	2.66 USD	[36,37]
c_Laboratory	Annual cost laboratory exams	1.28 USD	[36,38]
c_OAD	Annual average cost for OAD medication	24.85 USD	[36,38,39]
c_Insulin	Annual average cost for insulin therapy (weighted mean for monotherapy and insulin-OAD-combined therapy)	114.26 USD	[36,38,39]
c_Hospital	Annual average cost for inpatient treatment	35.89 USD	[40]

*adjusted.

**Table 2:** **Basic Parameters of Markov Model**

**Parameter**	**Source**	**Comment**
Prevalence rates	[1,5]	
Demography	[21,45,46]	
Mortality	[27,31]	
Age-specific incidence rates	[28]	Adjusted
Transition of stages	[29,32-35]	Adjusted
Diagnoses lag	[47]	Adjusted
Therapy options	[48]	Adjusted
Cost	[39,40]	Own calculations based on MoPoTsyo data from Phnom Penh

The mathematical prognosis follows a standard approach. The vector *w*_*t*_ represents the number of individuals in the specific health states (e.g. “healthy”, “undiagnosed, no complications”, “undiagnosed, complications”, “diet”, “OAD, no complications”, “insulin”, “OAD, complication”, “insulin, complication” “non T2DM death”, “T2DM death”) in time t. The respective vector *w*_*t+1*_ for the next period (*t + 1*) is calculated by multiplying the vector with the matrix of transition probabilities A [41], i.e.

$$ {\underset{\bar{\mkern6mu}}{w}}_{t+1}={\underset{\bar{\mkern6mu}}{w}}_t \cdot \mathrm{A} $$, with

$$ {\underset{\bar{\mkern6mu}}{w}}_t=\left(\begin{array}{c}\hfill {w}_1\hfill \\ {}\hfill \dots \hfill \\ {}\hfill {w}_n\hfill \end{array}\right);\cdot A=\left(\begin{array}{cccc}\hfill {a}_{11}\hfill & \hfill {a}_{12}\hfill & \hfill \dots \hfill & \hfill {a}_{1n}\hfill \\ {}\hfill {a}_{21}\hfill & \hfill {a}_{22}\hfill & \hfill \dots \hfill & \hfill {a}_{2n}\hfill \\ {}\hfill \vdots \hfill & \hfill \vdots \hfill & \hfill \ddots \hfill & \hfill \vdots \hfill \\ {}\hfill {a}_{n1}\hfill & \hfill {a}_{n2}\hfill & \hfill \dots \hfill & \hfill {a}_{nn}\hfill \end{array}\right) $$

Consequently, the number of individuals in the specific health states in time t is calculated as ${\underset{\bar{\mkern6mu}}{w}}_t={\underset{\bar{\mkern6mu}}{w}}_0 \cdot {\mathrm{A}}^t$ where *w*_*0*_ denotes the vector of the numbers of individuals in the conditions in the beginning of the simulation.

### Data

Table 2 exhibits the most important parameters and the sources. Where no country-specific data was available, we used international data and adjusted it to Cambodia. For this purpose we confronted Cambodian and international experts with the international data and asked them for their assessment of the relevance for Cambodia. With these estimates we firstly simulated the demographic and epidemiological development from 1988 to 2007. The results of these simulations were compared with the real figures from Cambodian statistics [42]. Finally, we adapted the parameters until the simulation fitted the reality to a best possible extent. This “calibration process” (e.g. [43]) was also used to adjust the transition of stages. As it is estimated that diabetes manifests about 10 years earlier in Asian countries than in Europe [44] the respective statistics had to be fitted. This standard approach of disease modelling under uncertainty calls for a set of sensitivity analyses which are presented in section Sensitivity analyses. The basic parameters are also expressed in the (Table 1).

The treatment costs are crucial so that detailed analysis was done based on the data of MoPoTsyo [36], data from WHO-CHOICE [37], the international drug price indicator guide [39] and additional research on market prices in Cambodia [38]. The costs of Metformin and of Glibenclamide were estimates as 25 US$ and 3 US$ p.a. p.c. With the assumption that 12% use mono-therapy of Metformin, 10% mono-therapy of Glibenclamide and 78% the bi-therapy the average cost per OAD-patient are about 25 US$. The cost per ml of insulin were estimated with 0.72 US$ [39]. At an average consumption of 10 ml we have annual cost of 86 US$ for insulin and 17 US$ for syringes. The combined therapy was estimated to cost 129 US$ p.a. p.c. Assuming that international standards [39] apply to Cambodia we reckoned that 57% of insulin requiring T2DM patients receive a monotherapy the average cost of insulin therapy is 114 US$ p.c. p.a. As far as not stated differently, all values are expressed in 2013 US$.

Based on these parameters we made a forecast of the epidemiology and economic impact of T2DM in Cambodia until 2028.

## Results

### Basic simulation

The basic simulation projects the epidemiological and economic consequences if the Government does not change its current policy, i.e., if the prevention and treatment of T2DM is not included in the basic health care package. In this case, only 12.5% of people requiring OAD and insulin can afford them by own funds or has access to them by charitable organisations. The basic simulation assumes that this rate remains stable.

**Figure 2:**
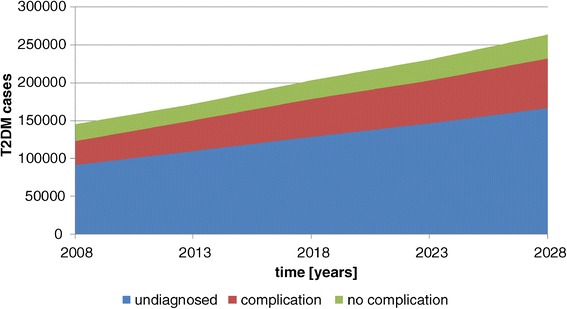
**T2DM cases – basic simulation.**

Figure 2 shows the development of diabetes in Cambodia under this condition. The number of people with T2DM is steadily increasing from 145,000 in the year 2008 to 264,000 in the year 2028 (+82%). At the same time the population of this age set increases by 67%, i.e., the prevalence rate increases from 4.0% to 4.4% in the age group ≥ 35 yrs.

The majority of T2DM cases remain undiagnosed. The rate is almost stable (63–64%) as the basic simulation assumes that no additional interventions are implemented. The number of diagnosed patients with complication, however, is increasing to a higher extent than the number of diagnosed patients without complications. In 2008 59% of diagnosed patients had complications, in the year 2028 this rate will have increased to 68%. This is due to the fact that the aging population and the longer duration of diabetes will result in more cases with complications.

**Figure 3:**
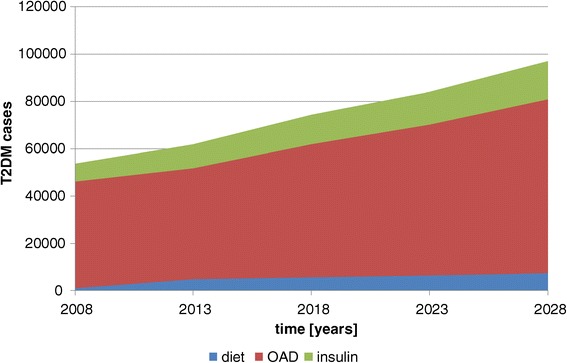
**T2DM therapies – basic simulation.**

Figure 3 shows the number of diagnosed T2DM cases requiring different forms of therapy. “Diet” means that a diagnosed patient does not receive any medication. Regularly he is obliged to live a specific diet and modify the life style. OAD are the next stage, followed by injection of insulin. In 2008, 2% would not require any form of medication, 84% would need OAD-therapy and 14% insulin. In the years 2028 8% of diagnosed cases will not require medication, 76% need OAD-therapy and 17% insulin therapy. Thus, a higher share of the population will be in the severe state of requiring insulin. The increase of 114% is much higher than the increase of the total number of diabetic cases in Cambodia. The decision-makers in Cambodia can expect that the worst medical and economic consequences of T2DM are still to come!

**Figure 4:**
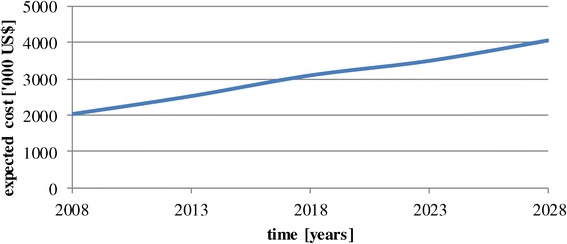
**Budget impact of T2DM – basic simulation.**

This is also reflected by the steadily increasing cost of treatment of patients with T2DM in Cambodia (Figure 4). In the moment, it is estimated that 12.5% of people requiring OAD- and insulin therapy do actually receive it [30,49]. The calculations assume that these figures remain stable. A scenario that accounts for increasing coverage with medication will be presented in section Oral Anti-Diabetic therapy.

In the year 2008 the 54,000 diagnosed T2DM patients would incur costs of 2 million US$ to cover all of diabetes treatment. 57% of this amount would have to be spent for OAD-therapy, the rest for insulin therapy. In the year 2028 this amount will have grown to 4 million US$ to cover 97,000 patients. If all patients (incl. non-diagnosed) had to be paid-for the respective figure would be 5.5 million US$ and 11 million US$. These calculations assume stable prices and no discounting. Thus, the costs will double due to a shift of patients towards the expensive patients with complications and requiring insulin. The expected average cost per diagnosed diabetic patient p. a. was 38 US$ in the year 2008 and will be 42 US$ in the year 2028.

The budget impact depends highly on the precision of the parameter estimates. Most critical is the estimate of the hospital cost. An increase of the cost by 50% leads to an increase of the cumulated cost by 40%, whereas the respective increase of the OAD-medication will result only in an increase of the total cost of 6%. Another critical parameter is the general and T2DM-related mortality. The earlier people die, the lower will be the cost. If the T2DM-related mortality increases by 20%, the cumulative cost will decrease by 16%. If the general mortality increases by the same rate, the respective decrease will be 17%. Even if we change all parameters towards the “best” possible case (according to estimates from the literature and from Cambodian experts), the budget requirement will increase until 2028.

In summary, Cambodia is facing an increasing number of diabetic patients requiring OAD- and insulin therapy. If the Government includes prevention and treatment of T2DM in the basic package, these costs will have to be refinanced by the Government even if the share of patients under treatment remains stable. Compared with the actual budget of the Ministry of Health these amounts seem not too high – but we have to keep in mind that they are additional to the existing expenditure for health care! Thus, there is a need to analyse the cost-effectiveness of interventions and select the best use of scarce resources.

### Screening programs

The basic simulation assumes that a patient is on average undiagnosed for seven years [47]. Consequently, treatment can only start late. At the same time, complications, early transition to higher states and diabetes-related death are higher than necessary. The purpose of screening programs is to determine T2DM cases as early as possible, ideally at the on-set of the disease to avoid these negative consequences. The impact of screening on the epidemiology and cost depends on the share of the population screened (e.g. only high-risk groups or total population) as well as on the sensitivity of the test. Without doubt, detecting every diabetic patient within one year will be technically impossible. However, in a first step we would like to present this utopic scenario of perfect screening, in the second step we will analyse the consequences of lower screening sensitivity and reduced coverage.

As first step we calculated the model for the case of the ideal screening program that detects every patient within one year after the on-set of T2DM. The consequences are an increase of the number of people living with T2DM and a strong decline of the number of T2DM-related death cases. In the year 2028, for instance, 275,000 people will live with T2DM instead of 264,000. The cumulative number of T2DM-related death cases from 2008 to 2028 will be 462,000 instead of 476,000. The share of diagnosed diabetics with complications sinks from 68% to 48%, and more diabetic cases need no medication (12% versus 8%). The total cumulative treatment costs increase from 61 to 68 million US$. However, the average cost per diagnosed diabetic patient of the year 2028 declines from 42 US$ to 33 US$.

The incremental cost-effectiveness ratio (ICER) compares the cost of an intervention with the effect (e.g. years of life saved, lives saved). An intervention is usually assessed as cost-effective if the cost per life-year saved (LYS) is less than the annual gross national product per capita (p.c.). In the case of Cambodia, an intervention is cost-effective if the ICER is less than 1,000 US$. If we assume the cost of urine sugar screening done by MoPoTsyo (i.e. 0.07 US$ p. c. plus 1 US$ for the confirmation test) the corresponding ICER is 464 US$/YLS, i.e., screening the entire population > 35 years annually for T2DM is cost-effective if the sensitivity is 100%.

In the second step we vary the coverage (share of population covered) and the sensitivity of the test. If we assume that 100% of the population are screened the cost per screening could be 1.05 US$ if the screening is 100% sensitive, 0.64 US$ if the sensitivity is 75% and 0.23 US$ if it were 50% sensitive. If the sensitivity goes below 36% screening will not be cost-effective at all.

**Figure 5:**
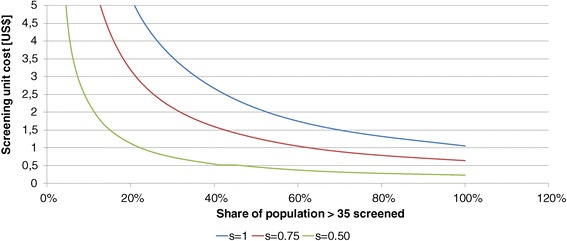
**Cost-effectiveness area of screening.**

If we focus on risk groups and reduce the coverage of the population the cost per screening could be higher. Every combination of coverage (x-axis) and cost (y-axis) to the lower-left of the curves of Figure 5 is cost-effective. If we limit, for instance, screening to 33% of the adult population ≥ 35 yrs. the cost per screening could rise to 3.18 US$ (sensitivity 100%), 1.93 US$ (sensitivity 75%) and 0.69 US$ (sensitivity 50%). For instance, limiting screening to the obese allows even higher unit costs while still safeguarding cost-effectiveness of the intervention. In the discussion we will address this issue again.

### Oral anti-diabetic therapy

OAD is not included in the basic package of health care services in Cambodia. Consequently, these drugs are only available for the very few protected by private health insurances or for those who can afford to pay for these drugs out-of-pocket. Consequently, it is estimated that only 12.5% of those diagnosed diabetics requiring OAD-therapy comply [30,49]. In this section we will analyse the consequences of improved access to OAD-therapy. This could, for instance, be possible if the Royal Government of Cambodia provides free or highly subsidized access to these drugs at rural health centres, i.e., if OAD medication becomes an element of the basic package of health care in Cambodia.

**Figure 6:**
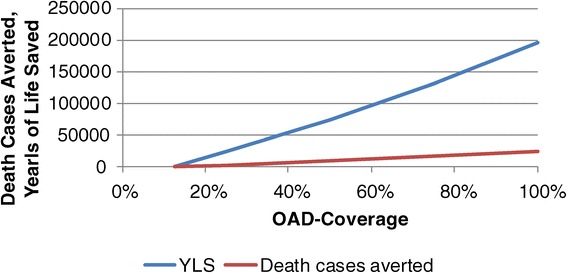
**Impact of different scenarios of coverage of OAD-therapy.**

Figure 6 exhibits the consequences of improved coverage of OAD-therapy on years of life saved and death cases averted. As expected, a higher degree of patients who require medication and receive medication leads to a higher number of years of life saved and death cases averted. The increase is almost linear. If 100% of patients requiring OAD-therapy receive it, 197,000 years of life are saved and 25,000 death cases are averted from 2008 to 2028. The percentage of diagnosed diabetics receiving OAD-therapy increases from 76% to 78%, but also the total number of people living with diabetes increases in all compartments of diagnosed diabetics. Thus, there are more people requiring insulin than before as more people survive the disease for a longer time. Consequently, the cost of diabetes treatment strongly increases, i.e., improving coverage of the population by OAD-therapy does not only require more resources to fund OAD-drugs but also to finance insulin for the higher number of patients who survived the OAD-phase. Consequently, the cost per diabetic patient increase from 42 US$ to 53 US$.

**Figure 7:**
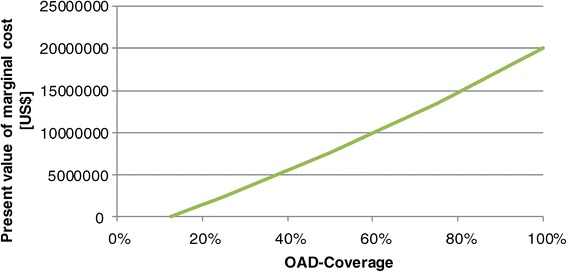
**Marginal cost of different scenarios of coverage of OAD-therapy.**

Figure 7 shows the present value of the marginal cost of improved OAD-coverage. The costs increase monotonously with increased coverage. However, the ICER is almost stable. An increase of coverage from 13% to 25% has an ICER of 102 US$ per YLS, an increase from 13% to 100% of 102 US$ (discounted with 5%). The respective figures for death cases averted are 841 (25%) and 780 US$ (100%) per death case averted, i.e., a higher coverage even improves the cost-effectiveness.

An ICER of 100 US$ per YLS is highly cost-effective, and this result does hardly depend on the interest rate. Even at a rate of 7.5% the ICER merely increases to 105 US$/YLS. However, cost-effectiveness does not imply feasibility as the budget constraints have to be respected. In the year 2028 Cambodia would have to spend 6.7 million US$ instead of 4 million US$ if all patients requiring OAD-therapy had access to this medication. A difference of 2.7 million US$ p. a. is significant for the National Budget.

### Insulin therapy

Insulin therapy in Cambodia is quite expensive and difficult to obtain. Consequently, it is estimated that only 12.5% of those requiring insulin therapy have access to it [30,49]. In the following we will analyse the consequences of improved access to insulin therapy.

**Figure 8:**
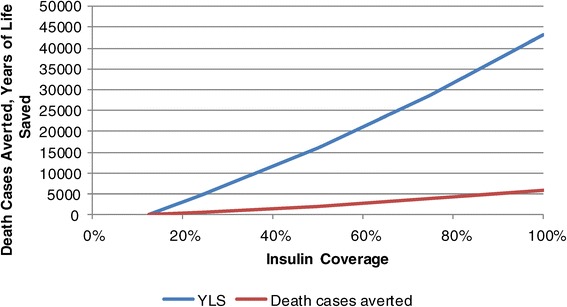
**Impact of different scenarios of coverage of insulin therapy.**

Figure 8 shows the impact of different coverage rates of insulin therapy on the number of death cases averted and the years of life saved. As expected, many death cases will be averted and many years of life saved if more diabetic patients requiring insulin have access to it. If all patients requiring insulin receive it 43,000 years of life will be saved and 5,800 death cases averted between 2008 and 2028. The number of people living with insulin therapy increases so that the share of diabetic patients with insulin therapy among all diagnosed diabetic patients increases from 17% to 22%. Consequently, the total cost increases from 42 US$ to 63 US$ per diagnosed diabetic case.

**Figure 9:**
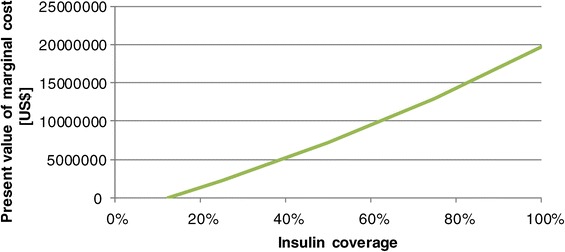
**Budget impact of different scenarios of coverage of insulin therapy.**

Figure 9 shows the present value of the additional cost caused by improved access to insulin for the period 2008 to 2028. If the insulin coverage increases from 12.5% to 25% the present value of these additional cost will be 2.3 million US$, if the entire population is covered it will be 20 million US$. Insulin therapy is quite expensive per person, so that the cost-effectiveness of improved access to insulin is lower than the cost-effectiveness of a higher coverage by OAD-therapy. The ICER is 451 US$ per YLS if the access improves from 12.5% to 25%. For an increase from 12.5% to 100% the respective figure is 457 US$ (r = 5%).

Consequently, improved access to insulin is cost-effective in Cambodia, but not as effective as improved access to OAD-therapy. From a purely economic perspective, improving OAD-therapy would have a higher priority than insulin therapy. However, patients requiring insulin are most likely suffering more than patients who can still deal with OAD-therapy. Thus, insulin therapy must have a high priority. However, the cost of improved access to insulin is considerable. If all patients requiring insulin are to receive full insulin coverage the additional cost of the year 2008 will be 2,473,319 US$, i.e., 6.5 million instead of 4.1 million US$. Cambodia will have to seek funds for this additional demand.

### Combined intervention

In most countries neither screening nor OAD- and insulin therapy are stand-alone interventions. A combined approach is seen best to reduce the burden of T2DM in Cambodia. Consequently, the last scenario analyses the consequences of introducing a “perfect” screening program and fully covering all patients requiring OAD- or insulin therapy.

The consequence of this optimal prevention and treatment approach is impressive: 582,000 years of life are saved and 78,000 death cases (−27%) are averted within the simulation period. The number of diagnosed diabetic cases increases from 37% to 90%. Among the diagnosed patients, the rate of those with complications declines from 68% to 51%. More patients do not require any medication (9% instead of 8%), but also more patients require insulin (21% instead of 17%).

However, substantial resources are needed to achieve these results. The annual budget for professional diabetes prevention and treatment in the year 2008 would be 8 million US$, i.e. 3.9 million US$ more than the current budget. This does not include the cost of mass screening. The model calculates that 6.1 million Cambodians of the respective age group (≥35 yrs.) will be living in Cambodia by then so that the costs of mass screening would be substantial. Assuming that the unit cost calculated for MoPoTsyo are representative for the entire country (0.07 US$ per screening unit) we could add 429,000 US$ just for screening. However, the ICER is still quite favorable. Without cost of mass screening it is 203 US$ per YLS (r = 5%), with mass screening 215 US$/YLS. Consequently, from a health economic perspective diabetes prevention and treatment is highly recommendable.

### Sensitivity analyses

The results of the basic simulation and the intervention analyses strongly depend on the reliability of the parameters. Until today the most serious problem of any modelling of (health) economic interventions in developing countries is the “the almost complete unreliability of what data is available and the absence of any recent data” [50]. Therefore, we conducted sensitivity analyses to assess the reliability of the predications. Parameters which were certain (e.g. fertility) were not included in the sensitivity analysis.

**Figure 10:**
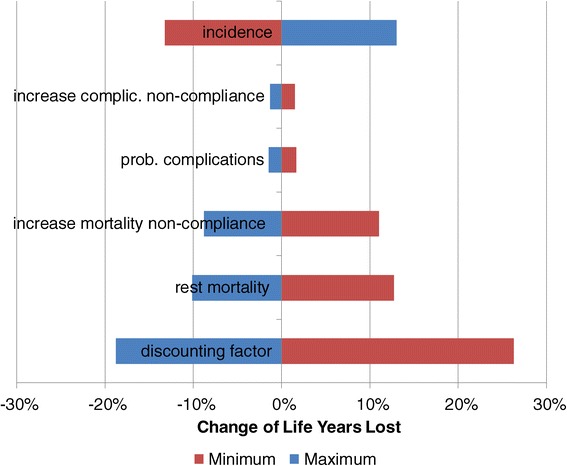
**Sensitivity of cumulative patient years.**

Figure 10 shows the impact of a change of parameters on the cumulative patient years (r = 5%). In absence of reliable minimum or maximum values of the parameters we increased or decreased all parameters by 20%. Merely the discounting factor was altered by ±50% (i.e. between 2.5 and 7.5%). Figure 10 includes only those parameters where the consequences of the respective increase or decrease are significant.

We realize that changes of the mortality and the incidence have a strong impact on the cumulative number of patient years. The incidence expresses the likelihood that a patient in the status “healthy” develops T2DM within a period. A change of this parameter will have a stronger impact on the epidemiology. Another important factor is the non-T2DM-related mortality (“rest mortality”). The higher the likelihood to die from any other cause of death, the lower is the number of patient years. In addition, patients who would require a certain treatment but cannot receive it will have a higher mortality (increase mortality non-compliance). The higher this increase, the lower is the cumulative number of patient years. All other variables have very limited impact on the patient years. It should also be noted, that all changes of parameters have under-proportional impact on the patient years.

**Figure 11:**
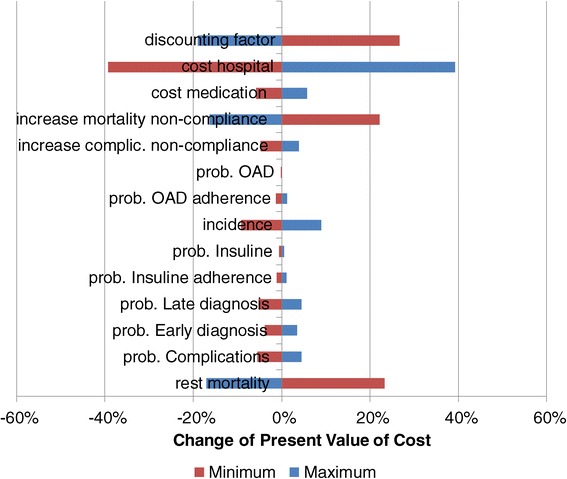
**Sensitivity of present value of cost.**

Figure 11 shows the consequences of a change of basic parameters on the present value of the cost of T2DM in Cambodia from 2008 to 2028. Again we included only those parameters where the increase or decrease had a significant influence. As expected, the hospital unit costs have the highest impact on the total cost of T2DM, but also the likelihood of developing diabetes (incidence) and the cost of medication (OAD and insulin) have a major impact. At the same time an increase of mortality (rest and increase due to non-compliance) and a higher discounting factor will decrease the present value of the cost. It becomes visible that the cost containment and the proper analysis of hospital accounts are crucial.

In addition, we calculated a “best-case” and a “worst-case” scenario as a multivariate sensitivity analysis. “Best” here means that all parameters (except the discounting factor) are altered by 20% so that the cumulated patient years are higher and costs are lower than in the basic scenario. “Worst” means an increase or decrease in the other direction. Table 3 shows the results. The range between the “best” and “worst”-case scenario is tremendous. If we estimate for all parameters the “worst” value the present value of cost will be almost three times as high as in the basic scenario. Here it is important to note that wrong estimates of all epidemiological probabilities (e.g. mortality, incidence) are worse than a wrong estimate of unit costs, but a wrong estimate of all parameters is much worse.

**Table 3:** **Best- and worst-case scenario**

**Scenario**	**Variant**	**Present value of cost [US$]**	**Difference**
Basic		37,119,499	-
Worst-case	Probabilities	72,203,153	95%
	Unit cost	55,679,249	50%
	Probabilities and unit cost	108,304,730	192%
Best-case	Probabilities	18,691,093	−50%
	Unit cost	18,559,750	−50%
	Probabilities and unit cost	9,345,546	−75%

Consequently, there is a great need for further research on basic epidemiological parameters and unit costs. For the time being our estimates of the basic model and the interventions are better than anything else in the literature – but they have to be taken with some caution. However, it is very unlikely that all parameters will be wrong so that this data can be taken as a starting-point of discussion.

## Discussion

Type 2 Diabetes Mellitus is a major public health challenge in Cambodia. Currently 170,000 inhabitants are estimated to have the disease [5], but health planners have to expect higher rates in future. The Markov model calculates that the number of cases of T2DM was 172,000 in the year 2013 and will grow to 264,000 in the year 2028. Based on the population ≥ 35 yrs. this will correspond to a prevalence rate of 4%. There is no doubt that the Ministry of Health, the Civil Society and health partners will have to invest effort to prepare for this “epidemic” of T2DM in Cambodia.

IDF [51] projects 364,000 diabetic cases in the year 2030 for Cambodia, but their model is rather simplistic in comparison to our Markov model. However, we have to accept that our forecast might underestimate the reality to come as we assume that transition probabilities remain constant. This means, for instance, that the likelihood of developing T2DM at a certain age remains stable through the simulation period. This assumption might be wrong if nutritional habits and physical exercise change. With the availability of processed food even in rural areas and the reduction of the share of population engaged in physical work it might be that our estimates turn out to be too conservative.

However, even at given transition probabilities the economic burden of T2DM for Cambodia is tremendous. Chronic complications are the main cost driver, and their number is increasing with time. The model calculates that 65,711 cases would require additional treatment for chronic complications in the year 2028. This results in a strong increase of cost from 2008 to 2028, i.e. from 2 million US$ to 4 US$. This refers to average annual cost per diabetic case of 42 US$.

Prevention and treatment of T2DM is not only a financial problem. Currently, the majority of health care workers in rural health centres are not well trained in the detection of cases and treatment of patients. Cambodia will have to invest effort to train their doctors, nurses and other health professionals in the prevention and treatment of NCDs, such as diabetes. As the numbers increase, there is an urgent need to intensify the existing training programs and tailor them even more towards NCDs.

At the same time Cambodia has to safeguard a stable supply of OAD-medication and insulin (including syringes etc.). This as well is not only a financial challenge. Cambodia is a tropical country where temperatures easily go up to 40 degrees Celsius. Thus, insulin requires an uninterrupted cooling chain. For most people in rural areas refrigerators are an unaffordable luxury. Providing community cooling systems for people living with diabetes (e.g. one per village) could be discussed. However, logistics will remain an issue.

The cost estimates for diabetes treatment differ widely within studies from South East Asia. Andayani & Imaningsih estimated a figure of 239.64 US$ p. a. for one Indonesian hospital, Andayani [11] calculated annual cost of 252.00 US$ for another hospital of this country. The direct cost which Ibrahim et al. [13] calculated for Malaysia (572.70 US$ p. a.) and Chatterjee et al. [17] for Thailand (555.33 US$) are even higher. However, the differences between these figures and the results of the simulation presented in this paper are the result of the very low cost of the Cambodian health system. Martin [52] found in her cost study of Cambodian public health services that the average cost of a health centre outpatient visit were 1.00 US$, the average inpatient case in the medical department of a district hospital (CPA-1) had costs of 20 US$. This is only a fraction of what it would cost in Thailand, Malaysia or Indonesia.

The low cost of Cambodian health services and the low financial resources required to prevent and treat T2DM in Cambodia reflect the different economic parameters of the respective countries. As Table 4 shows, Thailand and Malaysia are upper-middle income countries [53] with a national income per capita that is 5.5 and 11.1 (in US$) resp. 4.0 and 6.7 (in Int. US$) times higher. It is expectable that treating patients in these countries is much more expensive. Vietnam, Indonesia and the Philippines are lower-middle income countries. The GNP p. c. is also much higher. Laos and Myanmar would be much more comparable, but to our knowledge there is no data on the cost of diabetes in these countries.

**Table 4:** **Economic data of South-East Asian countries 2012**

	**Income classification**	**GNP [US$]**	**GNP [Int. US$]**	**Cost estimates [US$ p. a.]**	**GNP/cost**
Cambodia	Low	944	2330	42	22.5
Thailand	Upper-middle	5210	9280	555	9.4
Malaysia	Upper-middle	10432	16270	573	8.2
Vietnam	Lower-middle	1755	3620	-	-
Indonesia	Lower-middle	3557	4730	240-252	14.1-14.8
Philippines	Lower-middle	2587	4380	-	-

Source: [54].

As demonstrated in section Results the results of this simulation are highly sensitive to changes of the cost per service unit in hospitals. The model used the average cost per patient in medical departments of Cambodian hospitals as presented by Martin [52]. To our knowledge no cost analysis exists analysing the exact cost per diabetic inpatient case in Cambodia. This calls for further research.

In addition, the cost estimates of Martin reflect the situation of public health care providers. The costs of private providers are much higher. If we assume that private providers are included in diabetes care in Cambodia the cost will strongly increase and the cost-effectiveness of interventions will decrease. The Royal Government of Cambodia might make public-private partnership agreements to safeguard country-wide professional diabetes care while containing the costs.

The economic development of the country until 2028 will have two consequences. On one hand side, it is most likely that prices and wages will increase so that some of the cost estimates of this paper will be obsolete. However, the public budget will also increase. It is not yet determined whether the prices and wages of the health care sector will grow stronger or less than the public budget. If the prices and wages increase with the same rate as the gross national income but the national budget grows at a lower rate it is possible that the ICER of interventions remains constant but the feasibility to finance these interventions declines.

Our simulations show that improved access to OAD-medication and to insulin is cost-effective for Cambodia. The Royal Government of Cambodia should be advised to include professional treatment of T2DM patients in its basic package of health care services. Even a slight increase of coverage (from currently 12.5% to 25%) has already a tremendous impact on mortality and life years.

The cost-effectiveness of screening for T2DM depends on some parameters. Firstly, screening is cost-effective only if the sensitivity of the test is high enough. However, urine sugar tests might not be sufficiently precise as they might have a high number of false-negative cases in particular if made only once. They are inexpensive – but if the sensitivity is not at least 37% they will not be cost-effective even if we could limit the screening population to 1% of the adults. The estimates of the sensitivity of urine glucoses tests in the literature do not give a clear answer. Davies et al. estimated a sensitivity of 43% [55], Hanson et al. of 64% [56], Friderichsen and Maunsbach of 21% [57]. Other authors showed that the sensitivity differs strongly whether the test is done before or after glucoses intake [58]. Under the conditions of a Cambodian rural health centre, a urine blood sugar test is frequently quite unreliable. However, as the WHO rightly puts it: “Despite its low sensitivity, urine glucose testing may have a place in low resource settings where no other procedure is possible. This is particularly so, of course, when the prevalence of undiagnosed diabetes is likely to be high” [59].

**Figure 12:**
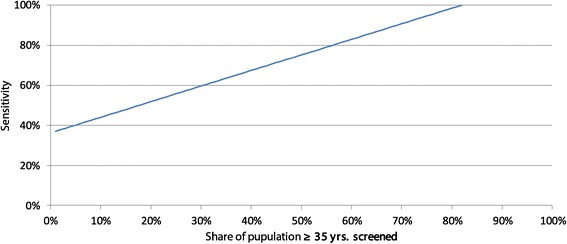
**Cost-effectiveness of screening with blood glucoses test.**

This indicates, secondly, that some concentration on risk groups must be sought to safeguard cost-effectiveness of screening. The risk factors obesity and low physical activity predict a high share of diabetic patients, but even focussing on only 10% of the population with a test with 50% sensitivity would not allow costs of more than 2.26 US$ per test to be cost-effective. Figure 12 shows the cost-effectiveness areas of screening with blood glucoses test assuming that one test costs 1.28 US$ [40]. All combinations to the lower-right of the curve are cost-effective.

## Conclusions

The simulations show that prevention and treatment of T2DM can be cost-effective even under the conditions of a least developed country like Cambodia. However, we must be aware that providing preventive and curative health care services at the point of provider is only one component of managing T2DM. Frequently undiagnosed diabetic cases will not show-up in health centres because they live too far away, have no transport or no time, or do not perceive any symptoms which would lead to a visit for diagnosis or therapy. At the same time, diagnosed patients might not comply with the instructions of the professional health care workers. Consequently, community-based approaches will be required that find potential patients, help them obtain professional help and improve compliance. The peer-educator network MoPoTsyo is such a community-based system that has shown the capability of strongly improving the situation of people living with T2DM. The Strategic Plan [22] foresees a nation-wide role of such networks in the fight against T2DM. The Ministry of Health could learn from the experiences of MoPoTsyo.

Diabetes is a great challenge to all stakeholders of the Cambodian health sector. It has financial, medical, demographic and social implications. However, the main challenge might be to develop another mind-set while addressing this chronic-degenerative disease. Until now, the entire health care system is based on the infection paradigm. One agent (e.g. plasmodium) causes one disease (malaria) and the connection between both is almost linear and deterministic. Consequently, seeking for the one cause, preventing or eliminating it is sufficient to provide proper health care. Chronic-degenerative diseases such as T2DM, instead, follow a multi-cause-multi-effect model, i.e., many risk factors and conditions contribute to their development (incl. a genetic risk). At the same time, the same set of risk factors might cause none or very different NCDs as no simple deterministic relationship between causes and effects exists. Consequently, a new focus on NCDs will require a new thinking about medicine, health care and the engagement of the health partners. Type 2 Diabetes Mellitus might be a door-opener to this different paradigm of health care in Cambodia.
